# Predicting the outcomes for out-of-hospital cardiac arrest patients using multiple biomarkers and suspension microarray assays

**DOI:** 10.1038/srep27187

**Published:** 2016-06-03

**Authors:** Chien-Hua Huang, Min-Shan Tsai, Kuo-Liong Chien, Wei-Tien Chang, Tzung-Dau Wang, Shyr-Chyr Chen, Matthew Huei-Ming Ma, Hsin-Yun Hsu, Wen-Jone Chen

**Affiliations:** 1 Department of Emergency Medicine, National Taiwan University Hospital, National Taiwan University Medical College, Taipei, Taiwan; 2 Division of Cardiology, Department of Internal Medicine, National Taiwan University Hospital, National Taiwan University Medical College, Taipei, Taiwan; 3 Graduate Institute of Epidemiology and Preventive Medicine, National Taiwan University, Taipei, Taiwan; 4 Department of Applied Chemistry and Institute of Molecular Science, National Chiao-Tung University, Hsinchu, Taiwan; 5 Division of Cardiology, Department of Internal Medicine, Lotung Poh-Ai Hospital, Yilan County, Taiwan

## Abstract

Predicting the prognosis for cardiac arrest is still challenging. Combining biomarkers from diverse pathophysiological pathways may provide reliable indicators for the severity of injury and predictors of long-term outcomes. We investigated the feasibility of using a multimarker strategy with key independent biomarkers to improve the prediction of outcomes in cardiac arrest. Adult out-of-hospital cardiac arrest patients with sustained return of spontaneous circulation were prospectively enrolled in this study. Blood samples were taken at 2 and 24 hours after cardiac arrest. Suspension microarray assays were used to test 21 different biomarkers. A total of 99 patients were enrolled, 45 of whom survived to hospital discharge. We identified 11 biomarkers that, when combined with clinical variables and factors of APACHE II score and history of arrhythmia, were independent determinants for outcome of in-hospital mortality (concordance = 0.9249, standard error = 0.0779). Three biomarkers combined with APACHE II and age were independent determinants for favorable neurological outcome at hospital discharge (area under the receiver-operator characteristic curve, 0.938; 95% confidence interval, 0.854 ~ 1.0). In conclusion, a systemic multiple biomarker approach using suspension microarray assays can identify independent predictors and model the outcomes of cardiac arrest patients during the post-cardiac arrest period.

The mortality rate is high during the post-cardiac arrest phase after initial return of spontaneous circulation. During the post-cardiac arrest period, the patient experiences ischemia-reperfusion injury and associated damage of global organs and systems^1^. Many pathophysiological reactions occur, which reflect the severity of injury in cardiac arrest and resuscitation events. These reactions interact with each other and evolve rapidly during the early post-cardiac arrest period^2^. Evaluating or measuring these biological changes can monitor organ damage and detect potential influences on the outcomes for cardiac arrest patients. Biomarkers from these biological reactions may be indicators of the severity of injury and predictors of long-term outcomes^3^. The outcome in cardiac arrest correlates with biomarkers from vital organs such as brain, heart, inflammatory system, and coagulation system^4^,^5^,^6^,^7^,^8^,^9^. However, the prediction values of single biomarkers are not high enough, and are not recommended as a decision tool in clinical practice. Lack of information about the interactions among crucial organs evolving with time leads to inconsistent data when using single biomarkers to predict outcomes^2^.

The concurrent measurement of multiple biomarkers is feasible due to technological advances during the proteomic era. Simultaneous measurement of biomarkers from different pathophysiological pathways in a small serum volume may enable the development of new biomarker strategies for cardiac arrest patients^10^,^11^. Suspension microarray is a new biotechnology that measures many different biomarkers involved in different pathophysiological responses during post-cardiac arrest syndrome^12^,^13^. Cardiac arrest is associated with high mortality and morbidity; therefore, early prediction of long-term outcomes is crucial for decisions of treatment strategies for cardiac arrest patients, because better management of post-cardiac arrest syndrome improves the outcomes of these patients^14^,^15^. The prediction of prognosis for cardiac arrest is still challenging for clinicians. Although current parameters of epidemiological characteristics, neurological reflexes, and electrophysiological responses improve predictions of outcome, the sensitivity and specificity of these parameters are not adequate and there is room for improvement^2^,^16^. Biomarkers related to diverse pathophysiological pathways are important because they can provide more information on real responses in organs and systems after cardiac arrest. In this study, we developed an integrated approach using bead-based suspension microarrays to analyse 21 selected plasma proteins from these diverse pathways to coordinately and rapidly evaluate multiple factors and pathways using the patient’s plasma. The results support the feasibility of using a multimarker strategy with key independent biomarkers to improve the prediction of outcomes in cardiac arrest.

## Results

### Baseline characteristics

**Figure 1:**
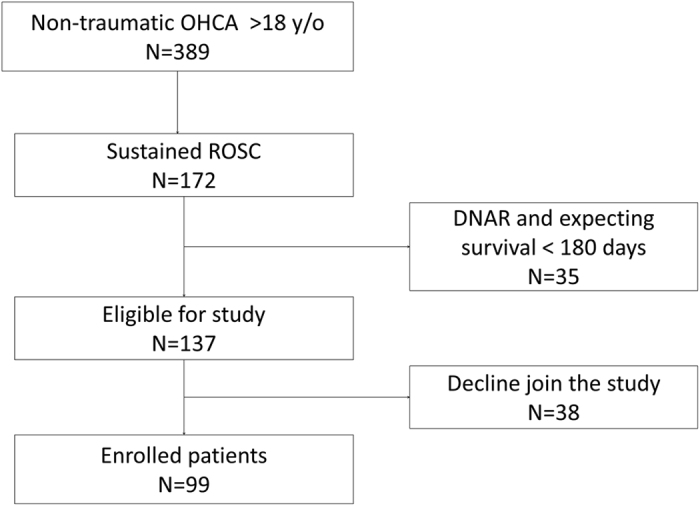
Enrollment of patients in the study.

During the study period, a total of 389 out-of-hospital cardiac arrest (OHCA) patients were identified, 172 of whom had sustained return of spontaneous circulation (ROSC) for more than 20 minutes. Thirty-five patients had do not attempt resuscitation orders and were excluded from the study; 99 of the remaining 137 patients gave informed consent for inclusion in the study (Fig. 1). Forty-five patients survived to hospital discharge. Shockable rhythm was more frequent as the initial rhythm in the surviving group compared to that of the deceased group (20.0% versus 9.3%, respectively; *p* = 0.092; Table 1). The highest acute physiology and chronic health evaluation II (APACHE II) scores within the first 24 hours after resuscitation were lower in the surviving group than in the deceased group (25.9 ± 7.2 versus 31.0 ± 8.8, respectively; *p* = 0.003).

### Circulating biomarker measurements using suspension microarray assays

Initial studies were performed to achieve appropriate sensitivity, specificity, and reliability in suspension microarray assays before the biomarkers were used for screening purposes. Cross-reactivity was assessed by incubating the multiplexed bead mixture with an analyte-specific detection antibody in the presence of a single analyte at a specific concentration (Supplemental Table S1). Intra- and inter- coefficient of variation (CV%) of the assays for the tested 21 plasma parameters are summarised in Supplemental Table S2. Of the 21 parameters, all had an intra-CV% within 10% and an inter-CV% below 20%. The recovery was calculated as the measured analyte concentration relative to the background concentration of the matrix plus the amount of spiked specific analyte (Supplemental Figure S1). As shown in Figure S1, most of the outliers appeared to have higher recovery (%) at the lower end of the concentration. Of 272 recovery measurements, 246 (90.4%) were within 80–120% of the acceptable recovery (%) range.

### Serum levels of circulating Nbiomarkers

The biomarker levels within 2 hours after ROSC (defined as at ROSC) in survivors and non-survivors were compared using univariate analyses (Table 2). Biomarkers with roles in inflammatory and anti-inflammatory reactions, including IL-8 and IL-10, were significant higher in non-survivors, while sCD40L was lower in non-survivors (Table 2). The brain-related biomarker S100B and the oxidative-stress related biomarker MDA-LDL were both significantly higher in non-survivors.

There were 63 patients with data on serum biomarker levels at 24 hours after ROSC, 18 cases refused further blood sampling for the study by the family, and 18 mortalities within the first day. The difference in serum biomarker levels at ROSC to 24 hour after ROSC (the biomarker level at 24 hours after ROSC minus the level at ROSC) were subjected to univariate analyses and compared between survivors (*n* = 40) and non-survivors (*n* = 23) as shown in Table 3. The inflammatory-related biomarker MCP-1 had higher difference levels in the non-survivor group than in the survivor group (−1,687.71 ± 4,100.62 versus 792.35 ± 4,253.81 pg/mL, respectively; *p* = 0.026).

### Biomarkers related to in-hospital mortality within the first 24 hours

There were 18 mortalities out of 99 patients within 24 hours after cardiac arrest and resuscitation. We analysed the independent predictors from all baseline characteristics, resuscitation variables, and biomarkers after power transformation for those with skewed distribution in the logistic regression model. We determined that serum levels of S100B (odds ratio 1.099, 95% CI 1.042–1.175), VCAM (odds ratio 15.202, 95% CI 2.805–120.427), PAI-1 (odds ratio 2.537, 95% CI 1.229–6.152), and IL-1β (odds ratio 1.299, 95% CI 1.043–1.657) were positively correlated with mortality within 24 hours after cardiac arrest (Table 4). By contrast, serum levels of adiponectin were negatively correlated with mortality within 24 hours after cardiac arrest (odds ratio 0.999, 95% CI 0.998–0.999, *p* = 0.0365). The area under the receiver operating characteristic (ROC) curve for the model was 0.885, with 95% CI 0.798–0.971.

### Independent predictors related to in-hospital mortality of patients who survived longer than 24 hours after resuscitation

We determined serum levels of biomarkers at ROSC and at 24 hours after resuscitation for patients surviving more than 24 hours. The difference in serum levels of a specific biomarker was defined as the level after 24 hours minus the level at ROSC. The multiple Cox’s proportional hazards model analyses indicated positive correlation with in-hospital mortality for the following biomarker levels: NT-proBNP difference, Thrombomodulin difference, MDA-LDL at ROSC, and soluble RAGE (sRAGE) difference (Table 5). The serum levels of S100B at ROSC, Cystatin-C at ROSC, sRAGE at ROSC, and VCAM at ROSC had non-linear distributions; therefore, we chose appropriate cutoff values for these biomarkers in the generalised additive model (GAM; Supplemental Figure S2) to show correlations with in-hospital mortality. The natural logarithms of the serum levels of Cystatin-C at ROSC (8.011–6.727) and sRAGE at ROSC (7.194–4.938) were associated with higher incidence of in-hospital mortality. The natural logarithm of the serum level of VCAM at ROSC less than 5.288 or more than 6.383 was associated with higher probability of in-hospital mortality. The natural logarithm of the serum level of IL-6 at ROSC were negatively correlated with in-hospital mortality. Two covariates, “history of cerebral vascular disease” and “APACHE II score”, violated the required proportional hazards assumption, and thus we stratified the Cox’s proportional hazards model by the “history of cerebral vascular disease” (yes versus no) and added the interaction term between the “APACHE II score” and “survival time (in days)” (i.e., APACHE II × Time) to the stratified Cox’s proportional hazards model. Moreover, as listed in Table 5, “APACHE II score × Time”, in the stratified Cox’s model suggested that after adjusting for the effects of the other covariates (including the stratified variable), the effect of “APACHE II score” on the hazard rate of in-hospital mortality would increase 1.004 times as survival time increased one day. Both concordance = 0.9249 > 0.7 and adjusted generalized R2 = 0.7593 > 0.15 indicated a very good fit of the stratified Cox’s regression model to the survival data.

### Independent predictors related to favorable neurological outcome at discharge

Independent predictors for favorable neurological outcome were evaluated using logistic regression analysis. Serum levels of S100B difference (odds ratio 0.998, 95% CI 0.996–1.000) and MPO at ROSC (odds ratio 0.433, 95% CI 0.197–0.763) were negatively associated with favorable neurological outcome as shown in Table 6. Better neurological outcome at discharge was associated with the natural logarithm values of 5.660–6.546 for the serum level of VCAM at ROSC, after choosing the appropriate cutoff values for this biomarker in the GAM model (Supplemental Figure S3). Increasing age (odds ratio 0.836, 95% CI 0.718–0.923) and APACHE II score (odds ratio 0.827, 95% CI 0.683–0.999) were associated with poorer neurological outcome at discharge. The area under the ROC curve for the model was 0.938, with 95% CI 0.854–1.

## Discussion

Complex pathophysiological processes occur after cardiac arrest and resuscitation. Post-cardiac arrest syndrome causes widespread disturbance of many reactions, organs, and systems. The intensity of these complex responses reflects the severity of injuries in different organs during cardiac arrest and the post-cardiac arrest period. In this study, we used a multiple marker strategy combined with suspension microarray assays to investigate the association of 21 biomarkers with potential injuries and reaction cascades during the post-cardiac arrest period. The observed changes in biomarker levels were well-correlated with survival to hospital discharge and neurological outcomes, and provided insights into the key reaction cascades and pathophysiological processes involved in post-cardiac arrest syndrome.

Some studies report that single biomarkers correlate with or predict outcomes in cardiac arrest^4^,^17^. However, no single biomarker can provide a highly discriminating accuracy in predicting outcomes due to the heterogeneity of cardiac arrest patients and scenarios^15^,^18^. Multiple organ systems are involved in and affected by cardiac arrest and resuscitation. Although some markers have been found to correlate with clinical outcome in cardiac arrest and resuscitation, different markers may interact with each other. True and net effect of a biomarker could be masked or misinterpreted when only single biomarkers are considered to correlate with disease progression or outcome prediction in cardiac arrest patients. The application of multiple biomarkers could help elucidate the complex interactions among different organs and systems during and after cardiac arrest^10^. The combination of markers from different physiological systems can improve risk stratification in diseases such as sudden cardiac death, sepsis, and cerebral vascular insults^19^,^20^,^21^.

We tested whether a multimarker strategy using miniaturised and multiplexed assays could improve risk stratification for cardiac arrest patients during the early post-cardiac arrest period. We used suspension microarray assays, which enable the measurement of all biomarkers in a small sample volume (less than 50 μL) of serum. Multiplex bead-based suspension microarrays combined with bioinformatics tools can provide efficient protein profiling in complex human body fluids such as plasma, and achieve appropriate sensitivity and high-throughput applications^12^,^22^. By combining the independent biomarkers with clinical and resuscitation variables, strong predictive power was obtained for the outcome predictions for one-day survival, survival to hospital discharge, and favorable neurological outcome.

Vital signs and pathophysiological processes change rapidly during the early post-cardiac period. Biomarkers at a single time point reflect the static condition of patients at a particular time point, but do not provide information on dynamic changes in reaction cascades of many reactions^6^,^16^. These changes can provide information on the improvement or deterioration of specific physiological systems and cascades during the temporal progression of post-cardiac arrest syndrome. In the present study, we evaluated two time points after cardiac arrest: the early period after resuscitation, and 24 hours after resuscitation. We observed statistically significant changes in some important biomarker levels, which were reflected in the regression model. An understanding of the changes in biomarker levels improves the prediction accuracy for outcomes of cardiac arrest patients. Instead of using one single biomarker at one time point, the new strategy evaluates multiple biomarkers at different time points. The results indicate that clinical application of biomarker analyses is a promising prognostic tool for predicting the survival and outcome of cardiac arrest patients.

The severity of brain injury during ischemia-reperfusion is a key factor related to the outcome of cardiac arrest patients. The central nervous system is one of the most susceptible systems for ischemia-reperfusion injury. Treatments that limit the damage to and enhance the recovery of neurological function are crucial for long-term survival of cardiac arrest patients. The biomarker S100B levels increase during the early post-cardiac arrest period, and this provides a good marker for neurological outcome of cardiac arrest patients^4^,^23^. Our study shows that S100B level at ROSC is an independent predictor for one-day and in-hospital mortality. The increasing S100 B level from ROSC to 24 hours after cardiac arrest is negatively correlated to favorable neurological outcome.

Another biomarker related to all three tested outcomes is VCAM. The levels of soluble endothelial adhesion molecules increase as early as 2 hours after cardiac arrest and resuscitation^24^. The increase of endothelial adhesion molecules is related to endothelial dysfunction, but also could be related to endothelial-derived vasodilation after ischemia and reperfusion injuries^25^. Endothelium overactivation induces overwhelming activation of coagulation and cytokine cascades, and leads to systemic inflammatory responses or sepsis-like syndrome in cardiac arrest^8^,^26^. Conversely, the appropriate activation of endothelium-derived vasodilation helps microvascular perfusion after ischemia-reperfusion injury in cardiac arrest^27^. In this study, we found that both excessively high and excessively low VCAM levels were associated with in-hospital mortality and poor neurological outcome. This biphasic pattern suggests that the appropriate level of response of VCAM is crucial for balancing beneficial and detrimental pathophysiological responses during post-cardiac arrest syndrome.

The activation of pro-inflammatory cytokines and inflammatory reactions occurs after cardiac arrest^28^. A high level of IL-6 during the first three days is associated with higher mortality rate in cardiac arrest patients, although the mechanisms have not been defined^29^. In our study, we found that the IL-6 level was inversely correlated to in-hospital mortality when we considered several key pro- and anti-inflammatory cytokines, coagulation, and endothelial markers together in the regression analysis model. Recent studies showed that IL-6 had positive effects on cardiac contractility and could induce nitric oxide-dependent protection after ischemia-reperfusion injury^30^,^31^. IL-6 also has an obligatory role in ischemia preconditioning and cardioprotective pathways through mitochondrial protection^32^. The net effect of IL-6 can be evaluated by eliminating its downstream activation of cytokine cascades, which was achieved by integrating these factors into the regression model and analyzing them together in the study. Our observations of the protective effects of IL-6 and the negative correlation with in-hospital mortality suggest that future comprehensive studies should be performed to evaluate the roles of individual cytokines in cardiac arrest.

Oxidative stress is one of the key physiological responses in post-cardiac arrest syndrome, and appropriate management of oxidative stress can improve myocardial function and patient outcome in the cardiac arrest model^33^,^34^. We observed that the oxidative stress biomarkers MDA-LDL and MPO were significantly associated with in-hospital mortality and poor neurological outcome, respectively. Good cardiac performance is associated with survival after cardiac arrest, while myocardial dysfunction can recover gradually after the initial cardiac arrest insult^2^. The difference between NT-proBNP levels at ROSC and after 24 hours is well-correlated with in-hospital mortality, and is a useful biomarker for evaluating outcomes of cardiac arrest patients. Cystatin C is a useful biomarker for renal dysfunction and acute cardiac or cerebral ischemia. Higher Cystatin C levels after acute coronary syndrome or ischemic stroke are associated with large infarctions and poor outcomes, respectively^35^. However, the infarction area is larger in Cystatin C-knockout animals, and exogenous supplementation of Cystatin C can reduce ischemic injuries in a stroke model^35^,^36^. Our observation of an association between biphasic Cystatin C levels and in-hospital mortality can be explained by an analysis of ischemia-reperfusion injuries of heart and brain in cardiac arrest.

Our study investigated metabolic biomarkers that are rarely evaluated in cardiac arrest. We found that adiponectin and sRAGE are associated with patient survival outcomes. Increased sRAGE levels reflect the receptor of advanced glycation end products (RAGE) activity, which has an important role in cardiovascular and cerebrovascular disease^37^,^38^. sRAGE has been identified as a valuable biomarkers that increases during acute coronary syndrome and rapidly declines after reperfusion therapy^39^. However, sRAGE inhibited myocardial apoptosis following ischemia-reperfusion injury through the JAK/STAT3 pathway^40^. Although the underlying mechanisms are unclear, the novel identification of these metabolic biomarkers as independent predictors of survival outcomes in cardiac arrest patients can help to generate a hypothesis for investigating their roles in patients with post-cardiac arrest syndrome.

There are some limitations to this study. The choice of biomarkers was based on the pathophysiology of cardiac arrest and organs that are probably involved. Representative biomarkers were selected, although other biomarkers could have been tested for their significance in the multimarker test. However, technical challenges necessarily limited the number of potential biomarkers that could be tested in this first study. The results support the feasibility and accuracy of multimarker suspension microarray assays, and provide a map for future measurement of additional novel biomarkers. The number of cases was relatively small in this study. This could lead to recruitment bias for the enrolled patients because informed consent was required from patients or their surrogates. Despite these potential limitations, the study may be regarded as generating a hypothesis that can be verified in future larger-scale studies.

In summary, many pathophysiological processes occur in different organs and systems after cardiac arrest and resuscitation. These processes influence the outcomes of cardiac arrest patients. In this study, we demonstrated that an assessment strategy based on multiple biomarkers and suspension microarray analyses could be used to evaluate the complex pathophysiological responses that occur during post-cardiac arrest syndrome. Combining these biomarkers with known clinical factors that influence patient outcomes enables highly concordant prediction of survival and neurological outcome in the regression model for cardiac arrest patients.

## Patients and Methods

### Study setting and population

This study was approved by the National Taiwan University Hospital (NTUH) Research Ethics Committee were carried out in accordance with the approved guidelines. OHCA patients were prospectively enrolled from October 2007 to May 2011 from NTUH, which is a tertiary medical center with approximately 100,000 emergency department visits per year. The emergency medical system is a single-tiered, fire department-based BLS-defibrillator system in a metropolitan city. At least two emergency medical technicians equipped with automated external defibrillators attend each call^41^.

Eligible patients included adult (>18 years old) non-traumatic OHCA patients who were successfully resuscitated with sustained ROSC for more than 20 minutes. Patients were excluded if they were transferred to other hospitals for post-cardiac arrest care, or if their relatives or surrogates refused to participate in the study. Patients with do not attempt resuscitation orders and those expecting to survive for less than 180 days due to underlying diseases before the cardiac arrest also were excluded. All enrolled patients or their surrogates gave written informed consent for this study.

### Blood sampling and assessment biomarkers

Blood samples were collected from a peripheral vein using a heparinised sterile syringe at the following two time points: within two hours after ROSC (defined as at ROSC), and at 24 hours after cardiac arrest. The blood was centrifuged for 30 minutes at 4,000 rpm, and then separated into plasma, buffy coat, and red blood cells (RBCs). The plasma was aliquoted and then stored at −70 °C until further analysis. The multimarker panel test utilised a sample volume of 50 μL. Changes in serum levels of biomarkers were defined as the difference of serum biomarker level at 24 hour minus the level at ROSC.

### Patient data and outcome measurements

All of the medical history and cardiac arrest event variables were recorded according to Utstein style recommendations^42^, and all of the pre-hospital and hospital records were reviewed. The causes of OHCA were documented when the patients were discharged from or died in the hospital, and were classified as cardiac events, respiratory events, and others. APACHE II criteria were evaluated, and the highest scores within 24 hours were recorded. The primary outcome endpoint was defined as in-hospital mortality. Secondary outcome was favorable neurological outcome with cerebral performance category 1 or 2 at hospital discharge.

### Construction of bead-based suspension microarray

Twenty-one candidate proteins involved in inflammatory, cardiovascular, neurological, oxidative stress, coagulation, and metabolic pathways were selected to establish the suspension microarray (Supplemental Table S3). The following three multiplex assays were used for the measurement of these 21 candidate proteins: one cytokine 9-plex assay [interferon gamma (IFN-γ), interleukin-1 beta (IL-1β), IL-1 receptor antagonist (IL-1Ra), IL-6, IL-8, IL-10, monocyte chemotactic protein-1 (MCP-1), soluble CD40 ligand (sCD40L), and soluble IL-2Ra] supplied by Milliplex^®^ (Catalog number MPXHCYTO-60 K); one lipid-associated human cardiovascular disease (CVD) 6-plex panel [N-terminal pro-brain natriuretic peptide (NT-Pro-BNP), lectin-type oxidised LDL receptor 1 (LOX-1), malondialdehyde (MDA)-LDL, myeloperoxidase (MPO), and thrombomodulin (TM)] supplied by WideScreen^®^ (Catalog number BPHCVD04-6); and one in-house 7-plex CVD panel [adiponectin, Cystatin C, extracellular newly identified receptor for advanced glycation end products binding protein (EN-RAGE), soluble RAGE, plasminogen activator inhibitor-1 (PAI-1), vascular cell adhesion molecule-1 (VCAM-1), and S100B]. Commercially available matched-pair antibodies and recombinant human proteins were used. S100B antibody pairs and recombinant proteins were supplied by Merck KGaA (Catalog number HNDG4-36 K, Darmstadt, Germany), and all others were purchased from R&D Systems, Inc. The multiplex bead-based suspension microarray was constructed as described previously^13^,^22^. The limit of detection [minimum of detected concentration (MinDC)] of most assays revealed excellent sensitivity. Measurements were performed using a Luminex 200^TM^ analyser (Luminex, Austin, TX, USA) with a five-parametric fitting curve to convert median fluorescence intensities (MFIs) into concentration values (Supplemental Figure S4).

### Statistical methods

The R 3.2.1 statistical software (R Foundation for Statistical Computing, Vienna, Austria) was used in data analysis. Categorical data were expressed as frequencies and proportions (%) and continuous data were represented by means and standard deviations (SD). In univariate analysis, the distributions of categorical variables and continuous variables were compared between two groups by Fisher’s exact test and Wilcoxon rank-sum test respectively. Power transformations X q with the power ladder (q), including q = 0 (natural logarithm), q = 0.25, and q = 0.5 (square root), were applied to some of the continuous variables to make their skewed-to-the-right (or positively skewed) distributions more symmetric for robustness. Then, multivariate analysis was performed by fitting multiple logistic regression models and multiple Cox’s proportional hazards model for the binary outcomes of (1) dying within one day in all patients and (2) favorable neurological outcome at discharge in patients who did not die within one day and for the survival outcome of time to survival at discharge in patients who did not die within one day respectively. In multivariate analysis, all the available independent variables were considered, regardless of whether they were statistically significant in univariate analysis, to avoid the interference of under-fitting bias. Two-tailed p value ≤ 0.05 was considered statistically significant.

To ensure the analysis quality, basic model-fitting techniques for (1) variable selection, (2) goodness-of-fit (GOF) assessment, and (3) regression diagnostics and remedies were used in regression analyses. Specifically, the stepwise variable selection procedure (with iterations between the forward and backward steps) was applied to obtain the candidate final regression models. The significance levels for entry and for stay were set at 0.15 to reduce the chance of missing important covariates. Generalized additive models (GAMs) were fitted to examine nonlinear effects of continuous covariates and, if necessary, to choose appropriate cut-off point(s) for discretizing continuous variables during the variable selection procedure. The final regression model was selected by excluding individual covariates with p value > 0.05 one at a time until all regression coefficients were statistically significant non-zero. Next, the estimated area under the receiver operating characteristic (ROC) curve (also called the c statistic), adjusted generalized R2, and the Hosmer-Lemeshow GOF test were used to assess the GOF of a fitted logistic regression model, while the concordance (equivalent to the c statistic) and adjusted generalized R2 for a fitted Cox’s proportional hazards model. Finally, the statistical tools for regression diagnostics, including verification of proportional hazards assumption, residual analysis, detection of influential cases, and check for multicollinearity, were applied to discover any model or data problems.

## Additional Information

**How to cite this article**: Huang, C.-H. *et al.* Predicting the outcomes for out-of-hospital cardiac arrest patients using multiple biomarkers and suspension microarray assays. *Sci. Rep.* **6**, 27187; doi: 10.1038/srep27187 (2016).

## Supplementary Material

Supplementary Information

## Figures and Tables

**Table 1:** Baseline characteristics and resuscitation variables of cardiac arrest patients who did and did not survive to hospital discharge.

Parameter	Survivors (*n* = 45)	Non-survivors (*n* = 54)	*P* value
Age (years)	68.79 ± 15.24	72.61 ± 12.41	0.115
Gender (male)	29 (64.4%)	29 (53.7%)	0.556
Diabetes mellitus	13 (28.9%)	17 (31.5%)	0.732
Hypertension	20 (44.4%)	15 (27.8%)	0.147
Coronary heart disease	11(24.4%)	14 (25.9%)	0.543
Heart failure	5 (11.1%)	6 (11.1%)	0.914
Arrhythmia	5 (11.1%)	3 (5.6%)	0.336
Cerebral vascular disease	12 (26.7%)	6 (11.1%)	0.066
COPD*	7 (15.6%)	6 (11.1%)	0.583
End-stage renal disease	6 (13.3%)	4 (7.4%)	0.333
Witnessed collapse	36(80.0%)	37 (68.5%)	0.175
Shockable rhythm	9 (20.0%)	5 (9.3%)	0.092
Cardiac cause of collapse	11 (24.4%)	13 (24.1%)	0.843
Epinephrine use during CPR	37 (82.2%)	49 (90.7%)	0.227
CPR duration (min)	23.80 ± 15.85	26.20 ± 15.70	0.506
APACHE II score at admission	25.98 ± 7.27	31.00 ± 8.81	0.003
Hypothermia treatment	20 (44.4%)	15 (27.8%)	0.094
Serum creatinine (mg/dL)	2.51 ± 2.71	2.99 ± 7.68	0.923
Estimated GFR†	42.04 ± 24.49	42.32 ± 22.42	0.951
Survival > one day			
Favorable neurological outcome	14 (31%)	0	0.023

^*^COPD: chronic obstructive pulmonary disease.

^†^GFR: glomerular filtration rate.

**Table 2:** Serum levels of biomarkers at ROSC between the survivors and non-survivors.

Biomarker	Survivors (n = 45)	Non-survivors (n = 54)	P value
sCD40L (pg/mL)	7921.88 ± 8499.79	6069.07 ± 8134.92	0.001
IL-8 (pg/mL)	333.57 ± 640.56	1398.44 ± 3856.73	0.002
MDA-LDL (ng/mL)	492.70 ± 2943.99	2915.64 ± 12500.75	0.012
IL-10 (pg/mL)	705.93 ± 1749.59	1225.26 ± 2530.12	0.013
S100B (pg/mL)	607.08 ± 702.96	1128.31 ± 908.02	0.032
MPO (ng/mL)	1310.17 ± 2187.97	1398.88 ± 1607.37	0.053
IL-6 (pg/mL)	1522.96 ± 5482.94	6199.62 ± 15649.39	0.071
NT-Pro-BNP (pg/mL)	2133.89 ± 2706.39	2063.73 ± 2169.36	0.094
MCP-1 (pg/mL)	3773.98 ± 4026.73	4750.92 ± 5383.34	0.119
Adiponectin (ng/mL)	1294.24 ± 733.36	1263.01 ± 655.30	0.173
sRAGE (ng/mL)	3362.76 ± 6247.06	5244.68 ± 11268.78	0.262
Thrombomodulin (pg/mL)	12772.88 ± 9668.86	14128.03 ± 12486.17	0.264
Cystatin-C_1(ng/mL)	2766.66 ± 5317.38	2577.16 ± 4384.30	0.278
IFNγ (pg/mL)	10.79 ± 31.29	5.37 ± 10.16	0.367
PAI-1 (ng/mL)	32.57 ± 41.40	39.15 ± 49.06	0.410
sIL-2Ra (pg/mL)	168.50 ± 784.63	68.28 ± 140.23	0.440
LOX-1 (ng/mL)	5.14 ± 9.87	7.51 ± 19.67	0.446
EN-RAGE (ng/mL)	234.93 ± 513.72	228.06 ± 676.30	0.451
IL-1Ra (pg/mL)	529.01 ± 2480.62	182.24 ± 391.12	0.479
IL-1β (pg/mL)	72.51 ± 450.67	70.24 ± 420.20	0.507
VCAM (ng/mL)	343.62 ± 192.08	417.69 ± 262.22	0.757

**Table 3:** Difference (Diff) of serum levels of biomarkers at 24 hours between the survivors and non-survivors.

	Survivors (n = 40)	Non-survivors (n = 23)	P value
MCP-1_Diff (pg/mL)	−1687.71 ± 4100.62	792.35 ± 4253.81	0.026
NT-Pro-BNP_Diff(pg/mL)	532.10 ± 2038.37	3669.49 ± 8694.62	0.102
S100B_Diff(pg/mL)	−31.36 ± 990.35	367.51 ± 1347.68	0.183
Thrombomodulin_Diff(pg/mL)	−1888.76 ± 8800.74	1176.24 ± 9761.07	0.206
LOX-1_Diff(ng/mL)	1.28 ± 16.78	−5.09 ± 27.70	0.259
IL-8_Diff (pg/mL)	−135.16 ± 738.90	50.93 ± 449.96	0.278
IL-6_Diff (pg/mL)	−173.19 ± 7163.20	1643.20 ± 5113.34	0.291
EN-RAGE_Diff(ng/mL)	−80.72 ± 509.68	19.62 ± 96.86	0.355
PAI-1_Diff(ng/mL)	−4.83 ± 38.49	5.93 ± 56.38	0.372
IL-1Ra_Diff (pg/mL)	−206.31 ± 678.80	−94.35 ± 168.57	0.441
IL-1β_Diff (pg/mL)	−22.35 ± 129.54	−1.69 ± 7.69	0.449
Adiponectin_Diff(ng/mL)	−235.93 ± 414.12	−167.85 ± 340.85	0.506
MPO_Diff(ng/mL)	−459.50 ± 2178.36	−197.12 ± 1260.59	0.599
IL-10_Diff (pg/mL)	−657.15 ± 1855.69	−490.86 ± 1482.96	0.715
sRAGE_Diff(ng/mL)	−1763.83 ± 4933.16	−1283.43 ± 5621.44	0.725
VCAM_Diff(ng/mL)	−17.90 ± 179.30	−29.44 ± 201.79	0.815
Cystatin-C_Diff(ng/mL)	−990.89 ± 4443.30	−1250.08 ± 5214.82	0.835
sCD40L_Diff (pg/mL)	−3139.60 ± 10531.21	−3567.89 ± 7358.57	0.864
IFNγ_Diff (pg/mL)	−1.7464 ± 14.08	−1.29 ± 3.47	0.881
sIL-2Ra_Diff (pg/mL)	0.63 ± 213.52	6.65 ± 78.57	0.897
MDA-LDL_Diff(ng/mL)	−274.77 ± 1650.16	−272.92 ± 3370.85	0.998

**Table 4:** Independent predictors for mortality within 24 hours by multiple logistic regression analysis.

Covariate	Odds Ratio	95% Confidence Interval	*p* Value
S100B at ROSC (square root pg/mL)^a^	1.099	1.042–1.175	0.0016
VCAM at ROSC (ln ng/mL)^b^	15.202	2.805–120.427	0.0040
PAI-1 at ROSC (ln ng/mL)^c^	2.537	1.229–6.152	0.0207
IL-1β at ROSC (ln pg/mL)^d^	1.299	1.043–1.657	0.0237
Adiponectin at ROSC (ng/mL)	0.999	0.998–0.999	0.0365

^a^S100B at ROSC (square root pg/mL): square root value of serum S100B level at ROSC

^b^VCAM at ROSC (ln ng/mL): natural logarithm of serum VCAM level at ROSC

^c^PAI-1 at ROSC (ln ng/mL): natural logarithm of serum PAI-1 level at ROSC

^d^IL-1b at ROSC (ln pg/mL): natural logarithm of serum IL-1b level at ROSC

Goodness-of-fit assessment: adjusted generalized *R*^2^ (Nagelkerke’s *R*^2^) = 0.479 > 0.3, the estimated area under the Receiver Operating Characteristic (ROC) curve = 0.885 > 0.7 (95% CI: 0.798–0.971), and the modified Hosmer and Lemeshow goodness-of-fit *F* test *p* = 0.6827 > 0.05 (df = 9, 84), which all indicated a good fit.

**Table 5:** Independent predictors for in-hospital mortality by multiple Cox regression analysis stratified by history of cerebrovascular disease.

Covariate	Hazard Ratio	95% Confidence Interval	*p* Value
S100B at ROSC (square root pg/mL) >31.381^a^	114.786	15.145–869.986	<0.0001
VCAM at ROSC (ln ng/mL) <5.288 or >6.383^b^	13.557	2.691–68.308	0.0016
IL-6 at ROSC (ln pg/mL)^c^	0.481	0.312–0.742	0.0009
NT-proBNP difference (pg/mL)	1.259	1.097–1.443	0.0010
8.011 >Cystatin-C at ROSC (ln ng/mL)>6.727^d^	67.689	8.221–557.347	0.0001
Cystatin-C difference (ng/mL) <−298.821^e^	14.017	2.444–80.374	0.0030
7.194 > sRAGE at ROSC (ln ng/mL) >4.938^f^	27.656	5.248–145.739	0.0001
sRAGE difference (ng/mL)	1.154	1.020–1.307	0.0231
MDA-LDL at ROSC (ln ng/mL)^g^	1.378	1.157–1.641	0.0003
Thrombomodulin difference (pg/mL) >3527.921^h^	23.306	3.913–138.828	0.0005
PAI-1 difference (ng/mL)	0.968	0.946–0.990	0.0049
History of arrhythmia	0.053	0.004–0.749	0.0297
APACH II score × Time (days)^i^	1.004	1.001–1.007	0.0183

^a^S100B at ROSC (square root pg/mL) >31.381: square root value of serum S100B level at ROSC > 31.381.

^b^VCAM at ROSC (ln ng/mL) <5.288 or >6.383: natural logarithm of serum VCAM level at ROSC < 5.288 and >6.383.

^c^IL-6 at ROSC (ln pg/mL): natural logarithm of serum IL-6 level at ROSC.

^d^8.011 >Cystatin-C at ROSC (ln ng/mL) >6.727: natural logarithm of serum level of Cystatin-C at ROSC <8.011 and >6.727.

^e^Cystatin-C difference (ng/mL) <−298.821: serum level of Cystatin-C at 24 hour minus level at ROSC <298.821.

^f^7.194 >sRAGE at ROSC (ln ng/mL) >4.938: natural logarithm of serum sRAGE level at ROSC <7.194 and >4.938.

^g^MDA-LDL at ROSC (ln ng/mL): natural logarithm of serum MDA_LDL level at ROSC.

^h^Thrombomodulin difference (pg/mL) >3527.921: serum level of Thrombomodulin at 24 hour minus level at ROSC >3527.921.

^i^APACHE II score × Time (days): time-dependent interaction term in the Cox model indicated that the effect of “APACHE II score” on the hazard rate of in-hospital mortality would increase 1.004 times as survival time increased one day.

Goodness-of-fit assessment: adjusted generalized *R*^2^ = 0.7593 > 0.15 and concordance = 0.9249 (se = 0.0779), which indicated an excellent fit.

**Table 6:** Independent predictors for favorable neurological outcome by multiple logistic regression analysis.

Covariate	Odds Ratio	95% Confidence Interval of Odds Ratio	*p* Value
S100B difference (pg/mL)	0.998	0.996–1.0000	0.0447
6.546 >VCAM at ROSC (ln ng/mL) >5.660^a^	93.524	3.7950–2304.7773	0.0055
MPO at ROSC to the 0.25 power (ng/mL)^b^	0.433	0.197–0.763	0.0127
Age (year)	0.836	0.718–0.923	0.0040
APACHE II	0.827	0.683–0.999	0.0497

^a^6.546> VCAM at ROSC (ln ng/mL) >5.660: natural logarithm of serum VCAM level at ROSC >5.669 and <6.546

^b^MPO at ROSC to the 0.25 power: serum MPO level at ROSC to the 0.25 power

Goodness-of-fit assessment: adjusted generalized *R*^2^ (Nagelkerke’s *R*^2^) = 0.667 > 0.3, the estimated area under the Receiver Operating Characteristic (ROC) curve = 0.938 > 0.7 (95% CI: 0.854–1), and the modified Hosmer and Lemeshow goodness-of-fit *F* test *p* = 0.0857 > 0.05 (df = 9, 55), which all indicated an excellent fit.
