# Supplementary material for: Predicting the outcomes for out-of-hospital cardiac arrest patients using multiple biomarkers and suspension microarray assays
Source: Sci Rep. 2016 Jun 3;6:27187. doi: 10.1038/srep27187 (PMC4891702; doi:10.1038/srep27187)

**Supplementary Data**

**Predicting the outcomes for out-of-hospital cardiac arrest patients using multiple biomarkers and suspension microarray assays**

Chien-Hua Huang, MD, PhD^1,2^; Min-Shan Tsai, MD, PhD^1^; Kuo-Liong Chien, MD, PhD^3^; Wei-Tien Chang, MD, PhD^1^; Tzung-Dau Wang, MD, PhD^2^; Shyr-Chyr Chen, MD^1^; Matthew Huei-Ming Ma, MD, PhD^1^; Hsin-Yun Hsu, PhD^4,*^; Wen-Jone Chen, MD, PhD^1,5*^

**Supplemental Table S1.** (A) Cross-reactivity test in the 7-plex assay. The commercially available 9-plex cytokine panel and lipid-associated human CVD 6-plex assays were validated by the manufacturer, and cross-reactivity between all assays was < 1%. The newly developed 7-plex panel (adiponectin, cystatin C, EN-RAGE, sRAGE, PAI-1, VCAM-1 and S100B) was also examined, and no significant cross-reactivity was observed in the assay. The assays were performed using each analyte at a specific concentration of standards (listed in (B)) and incubated in the present of one detection antibody. “Max MFI” = maximal MFI signal generated in the reaction; “Blank MFI” = signal generated when no analyte was present; “Max. S/N” = maximal signal/noise ratio.

(A)

| **Detection Antibody** | **Analyte** | | | | | | |
| --- | --- | --- | --- | --- | --- | --- | --- |
|  | **Adiponectin** | **Cystatin C** | **EN-RAGE** | **sRAGE** | **PAI-1** | **VCAM-1** | **S100B** |
| **Adiponectin** | **9122** | 10 | 23 | 14 | 18 | 13 | 4 |
| **Cystatin C** | 5 | **4130** | 22 | 13 | 18 | 13 | 4 |
| **EN-RAGE** | 4.5 | 12 | **12688** | 13 | 18 | 12 | 5 |
| **sRAGE** | 5 | 12 | 23 | **9311** | 17 | 12 | 7 |
| **PAI-1** | 5 | 13 | 22 | 13 | **8562** | 13 | 5 |
| **VCAM-1** | 6 | 9 | 24 | 17 | 17 | **8222** | 7 |
| **S100B** | 4 | 10 | 23 | 16 | 15 | 14 | **9456** |
| **Max. MFI** | 9122 | 4130 | 12688 | 9311 | 8562 | 8222 | 9456 |
| **Blank MFI** | 4 | 12 | 22 | 13 | 16 | 11 | 3 |
| **Max. S/N** | 2280.5 | 344.2 | 576.7 | 716.2 | 535.1 | 747.5 | 3152 |

(B)

| **Analyte** | **Adiponectin** | **Cystatin C** | **EN-RAGE** | **sRAGE** | **PAI-1** | **VCAM-1** | **S100B** |
| --- | --- | --- | --- | --- | --- | --- | --- |
| **ng/mL** | **733** | **69** | **12** | **15** | **13** | **24** | **10** |

**Supplemental Table S2**. Intra-CV% , inter-CV%, R2 of standard curves, MinDC and MaxDC of the assays for the 21 investigated circulating biomarkers.

| Analyte | intra-CV(%) | inter-CV(%) | R2 | MinDC | MaxDC |
| --- | --- | --- | --- | --- | --- |
| IFNg (pg/mL) | 0.56 | 6.75 | 1.00 | 1.60 | 6241.50 |
| IL-1b (pg/mL) | 0.02 | 5.28 | 1.00 | 0.06 | 10644.19 |
| IL-1ra (pg/mL) | 0.19 | 13.01 | 0.99 | 0.76 | 13455.05 |
| IL-6 (pg/mL) | 0.31 | 8.79 | 1.00 | 0.39 | 11911.33 |
| IL-8 (pg/mL) | 0.32 | 10.94 | 1.00 | 0.95 | 12402.52 |
| IL-10 (pg/mL) | 0.54 | 11.39 | 1.00 | 1.32 | 9364.45 |
| MCP-1 (pg/mL) | 1.24 | 13.12 | 1.00 | 1.37 | 25041.99 |
| sCD40L (pg/mL) | 0.07 | 4.68 | 1.00 | 0.06 | 21747.28 |
| sIL-2Ra (pg/mL) | 0.06 | 11.29 | 1.00 | 0.07 | 10318.51 |
| LOX-1 (ng/mL) | 8.80 | 15.73 | 1.00 | 0.08 | 26712.82 |
| MPO (ng/mL) | 0.47 | 15.47 | 0.98 | 5.27 | 28077.97 |
| MDA-LDL (ng/mL) | 4.48 | 17.88 | 1.00 | 1.45 | 24645.17 |
| Thrombomodulin (pg/mL) | 5.67 | 12.11 | 0.99 | 48.38 | 181196.97 |
| NT-Pro-BNP (pg/mL) | 2.43 | 9.75 | 1.00 | 1.72 | 7940.40 |
| Adiponectin (ng/mL) | 0.82 | 10.81 | 1.00 | 0.68 | 932.36 |
| EN-RAGE (ng/mL) | 0.60 | 10.65 | 0.99 | 0.01 | 31.89 |
| PAI-1 (ng/mL) | 1.11 | 17.21 | 1.00 | 0.03 | 3.99 |
| Cystatin-C (ng/mL) | 9.55 | 16.45 | 0.94 | 0.09 | 17.49 |
| VCAM-1 (ng/mL) | 0.61 | 10.75 | 1.00 | 0.01 | 23.86 |
| sRAGE (pg/mL) | 0.84 | 8.14 | 1.00 | 7.35 | 26130.24 |
| S100B (pg/mL) | 1.26 | 11.25 | 0.99 | 2.55 | 10962.84 |

**Supplementary Table S3:** List of screened 21 biomarkers in suspension microarray assays

| **Inflammation-related biomarkers** | **Endothelium and Coagulation-related** |
| --- | --- |
| Interferon gamma (IFNg) | Thrombomodulin (TM) |
| Interleukin (IL)-1beta | Plasminogen activator inhibitor-1 (PAI-1) |
| IL-1 receptor antagonist (IL-1ra) | Vascular cell adhesion molecule-1 (VCAM-1) |
| IL-6 |  |
| IL-8 | **Oxidative stress-related** |
| IL-10 | Lectin-type oxidized LDL receptor 1 (LOX-1) |
| Monocyte chemotactic protein -1 (MCP-1) | Malondialdehyde (MDA)-LDL |
| soluble CD40 Ligand (sCD40L) | Myeloperoxidase (MPO) |
| soluble interleukin-2 receptor antagonist (sIL-2 Ra) |  |
|  |  |
| **Cardiac and renal -related** | **Metabolism-related** |
| Cystatin C | Adiponectin |
| N-terminal pro- brain natriuretic peptide (NT-Pro-BNP) | Extracellular newly identified receptor for advanced glycation end products binding protein (EN-RAGE) |
| **Brain-related** | soluble RAGE (sRAGE) |
| S100B |  |

**Supplemetary Figure S1.** Recovery was calculated as the measured analyte concentration relative to the background concentration of the matrix plus the spiked amount of the specific analyte. Most of the outliers appeared to have higher recovery (%) at the lower end of the concentration.


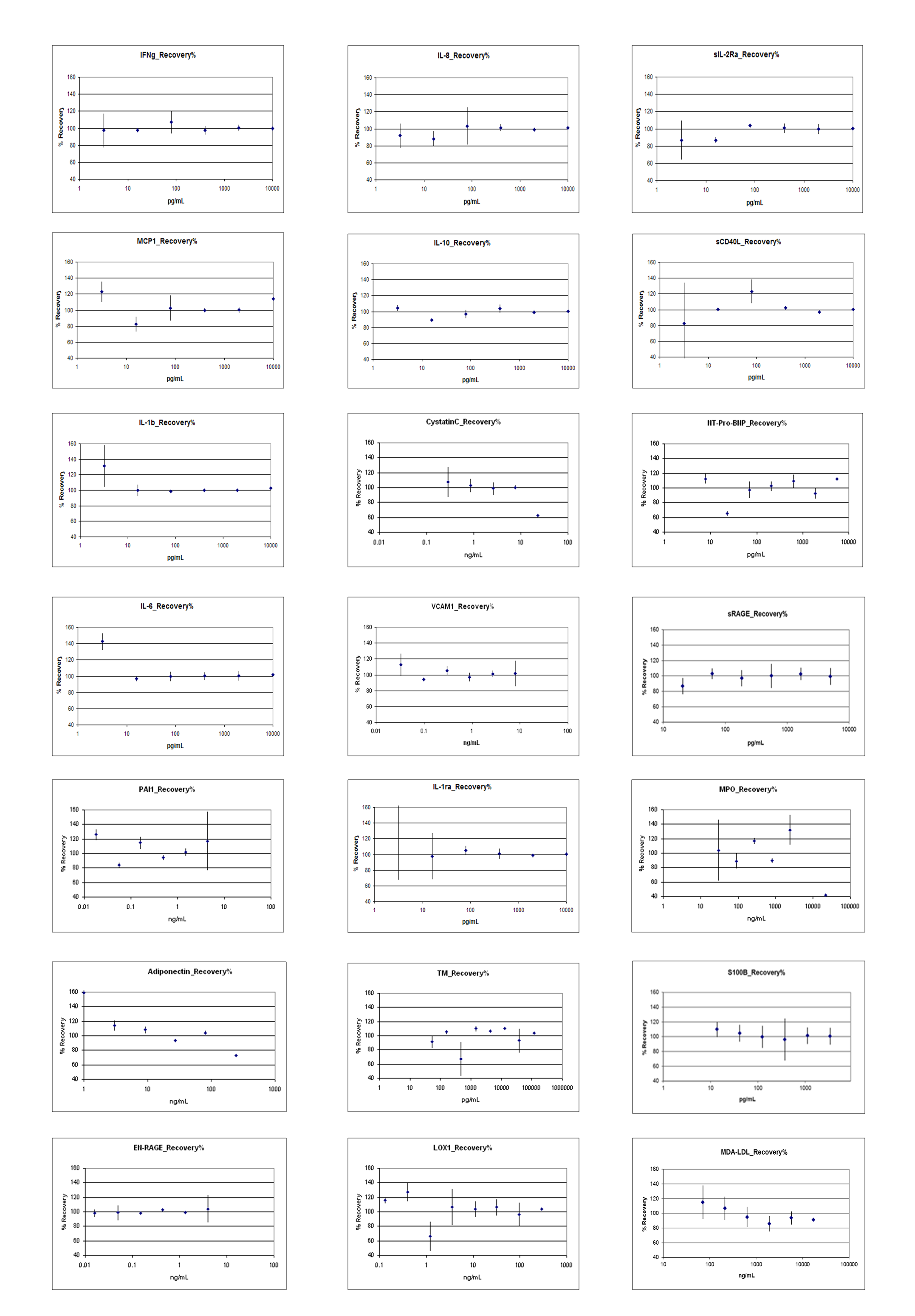


**Supplemetary Figure S2.** Generalised additive models (GAMs) plot and the appropriate cut-off point(s) for dichotomizing a continuous variable during the variable selection procedure for in-hospital mortality.

Figure S2a:GAM plot for S100B at ROSC


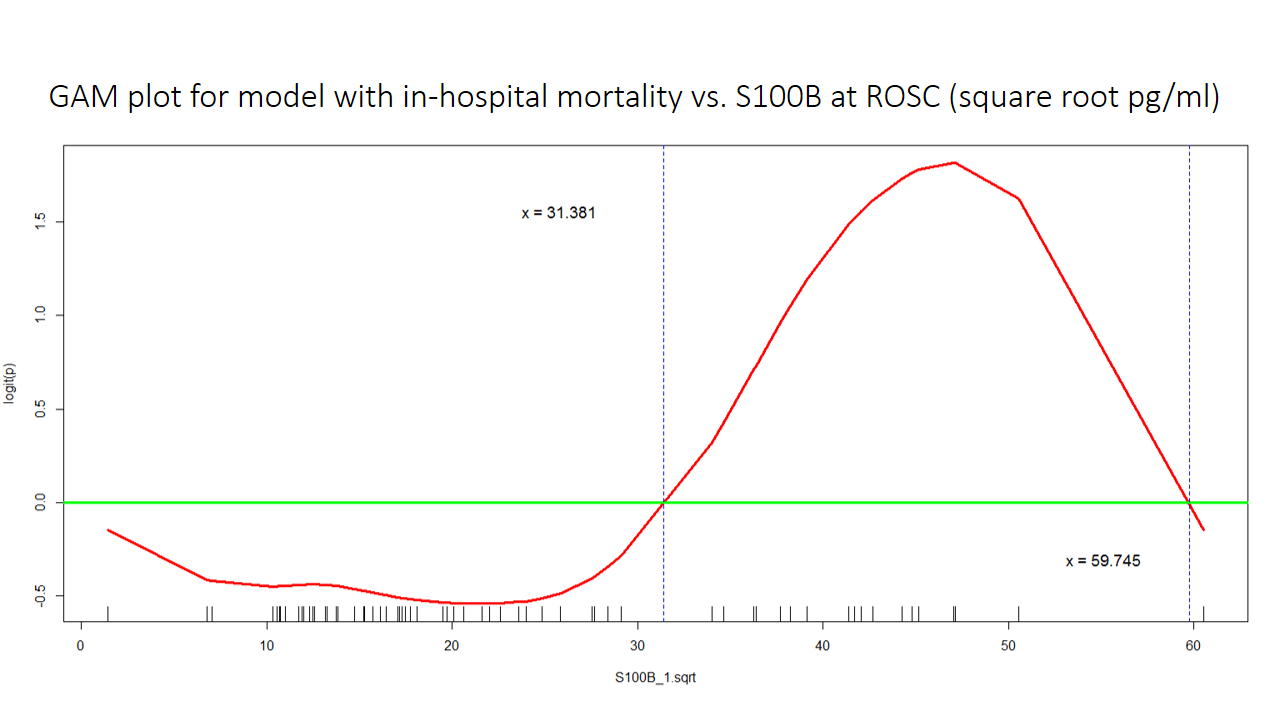


Figure S2b:GAM plot for VCAM at ROSC
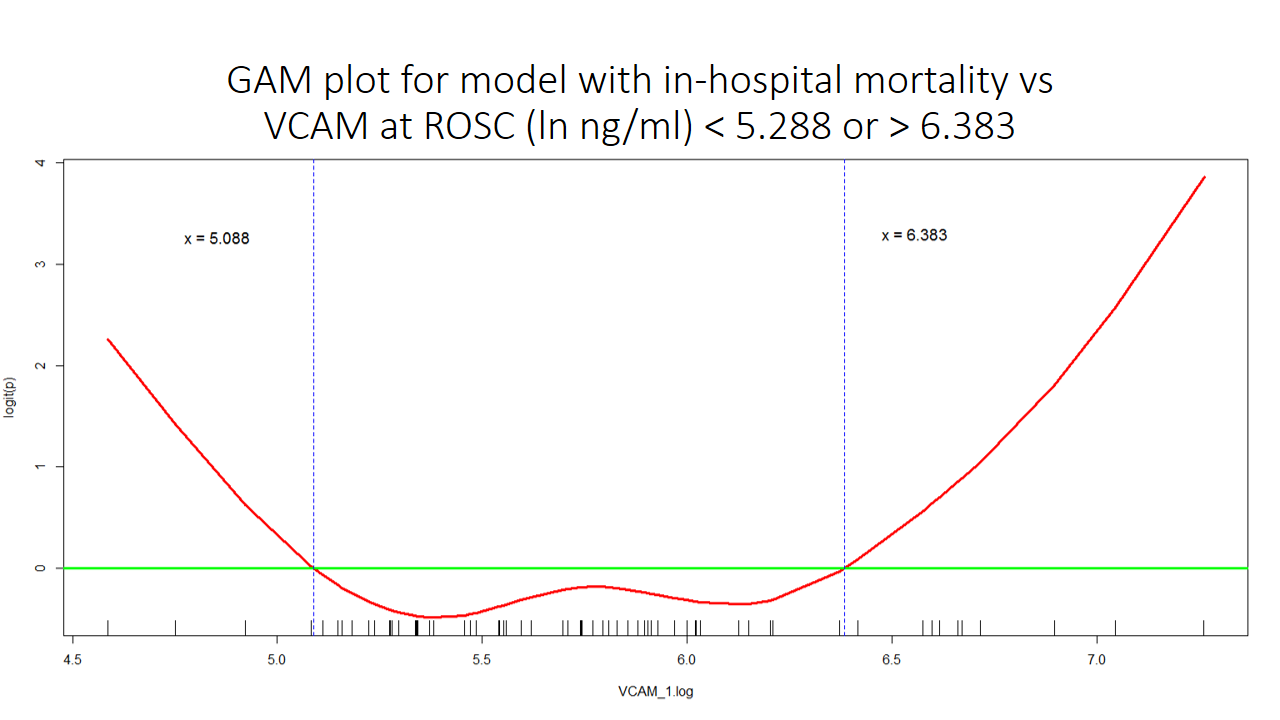


Figure S2c: GAM plot for Cystatin-C at ROSC
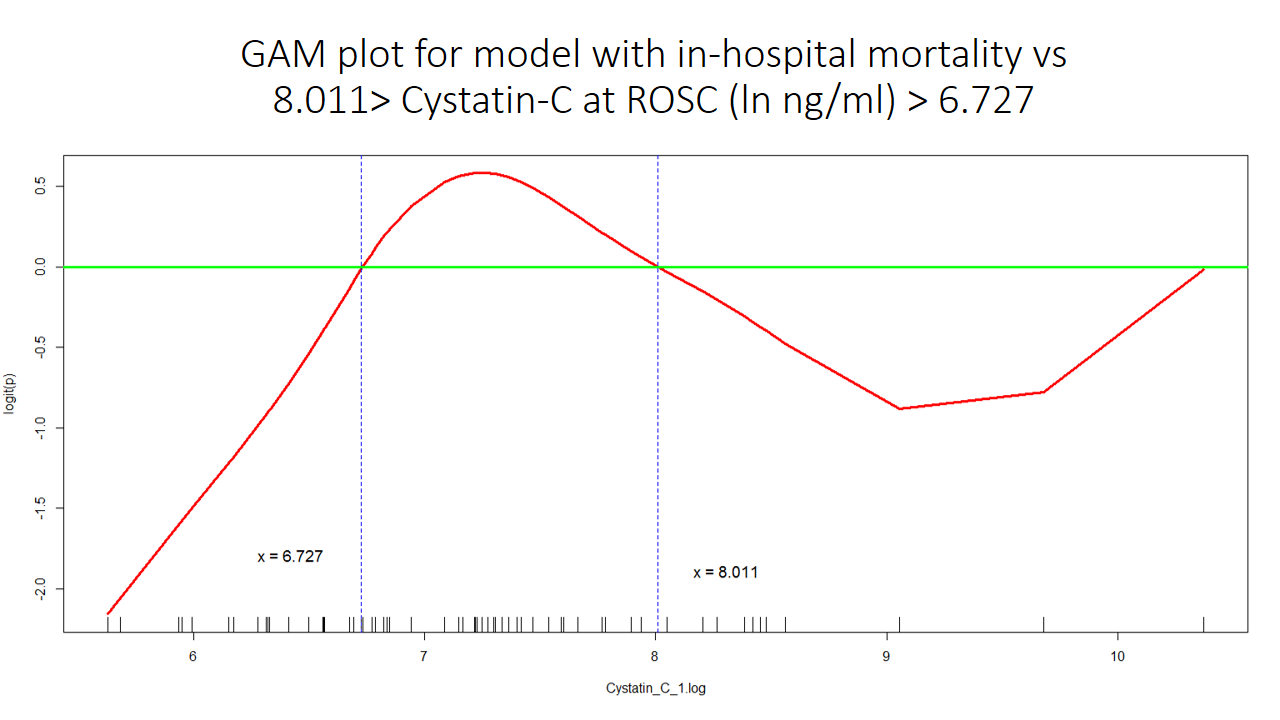


Figure S2d: GAM plot for Cystatin-C difference


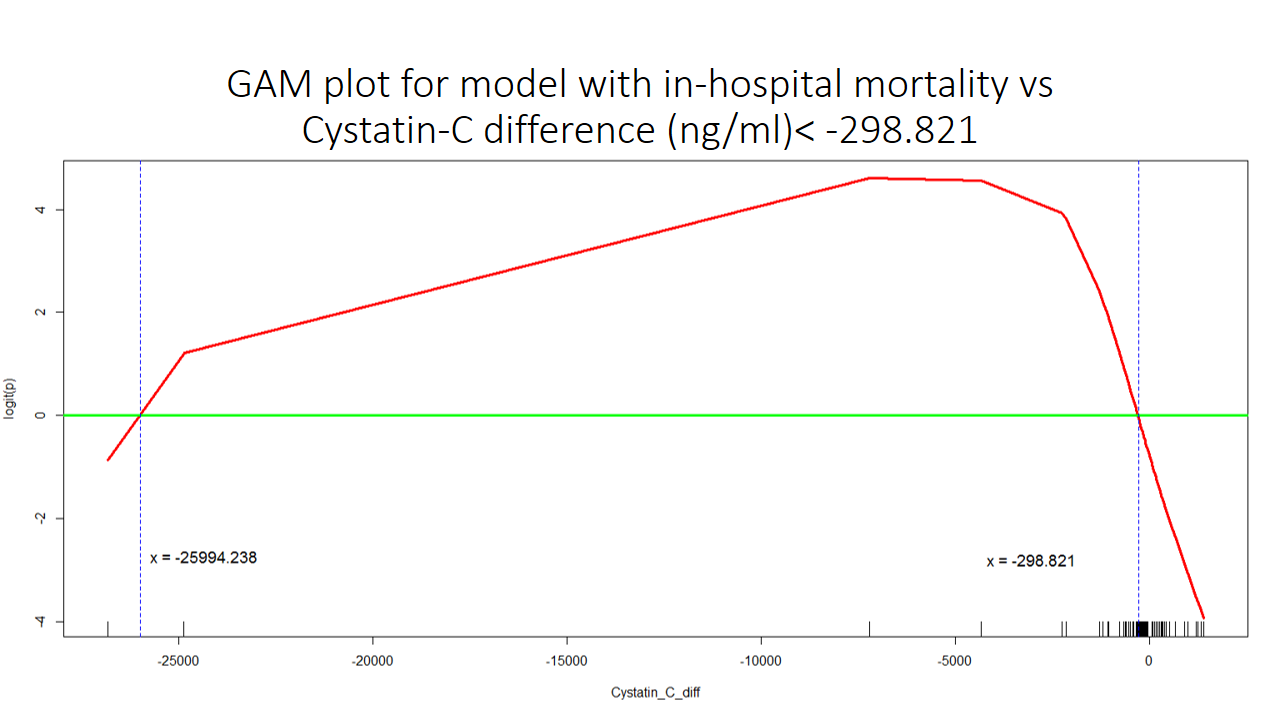


Figure S2e: GAM plot for sRAGE at ROSC
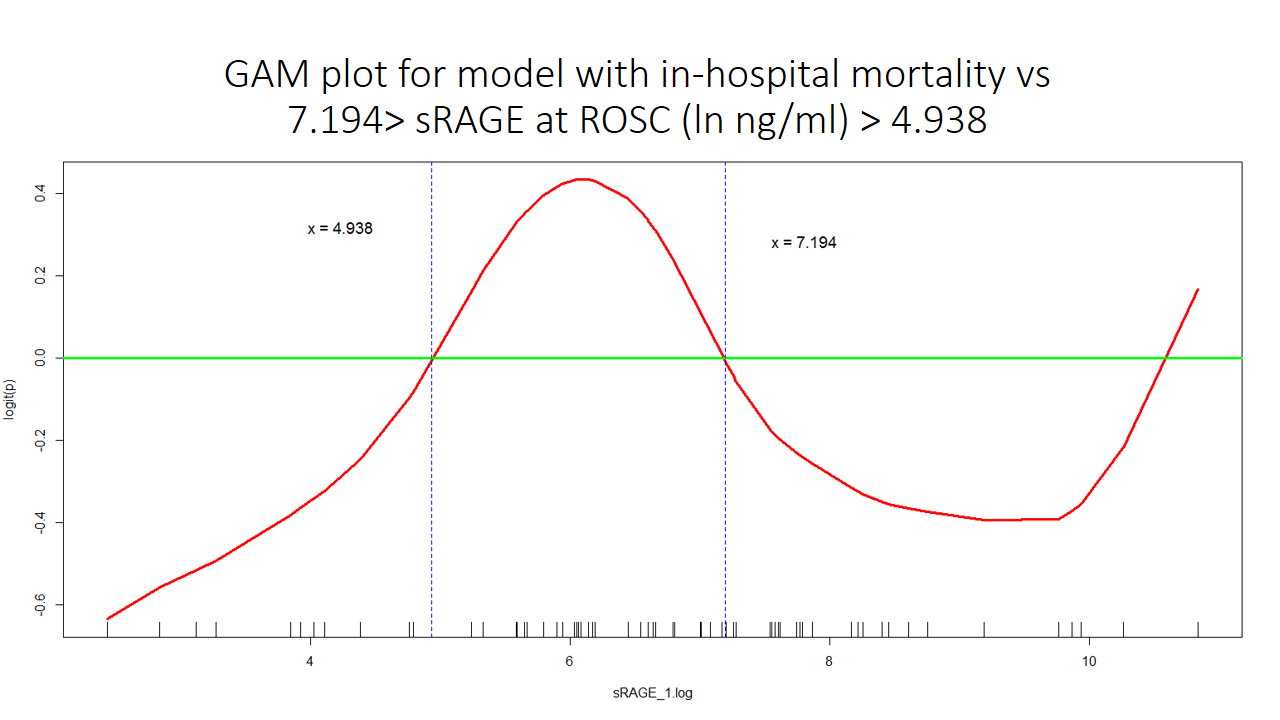


Figure S2f: GAM plot for Thrombomodulin difference


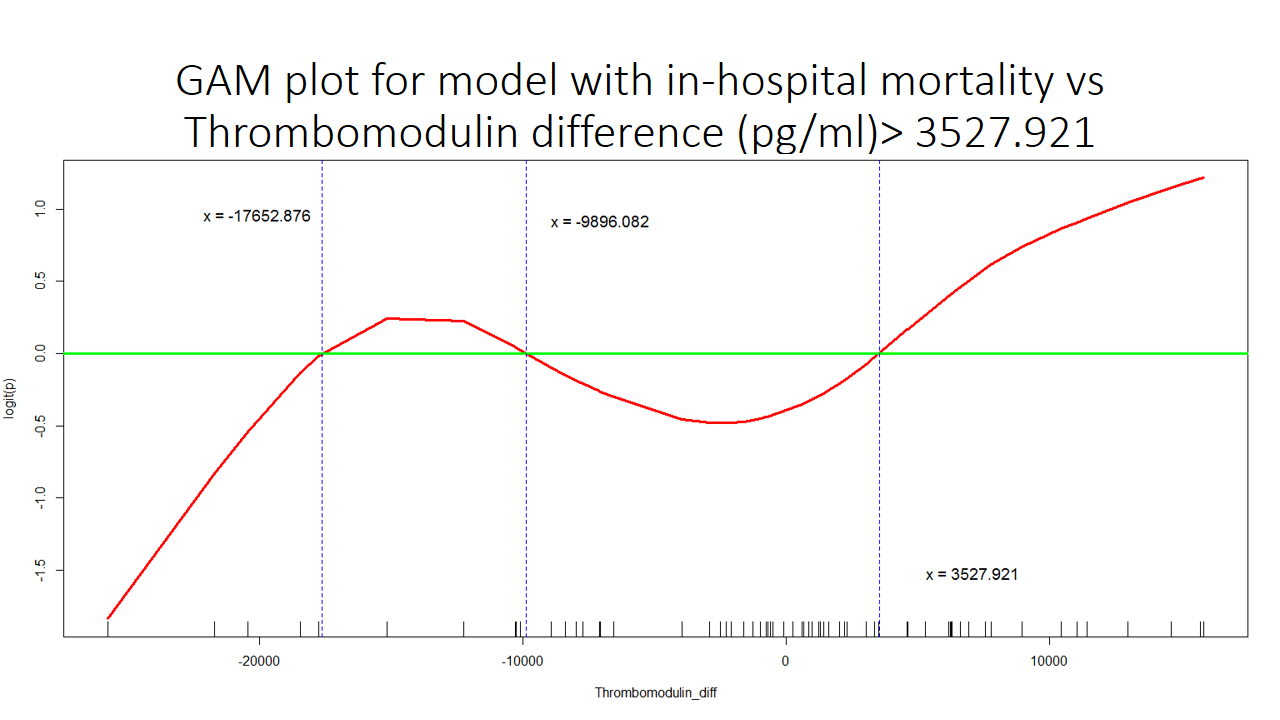


**Supplementary Figure S3:** Generalised additive models (GAMs) plot and the appropriate cut-off points for dichotomizing VCAM during the variable selection procedure for favorable neurological outcome.


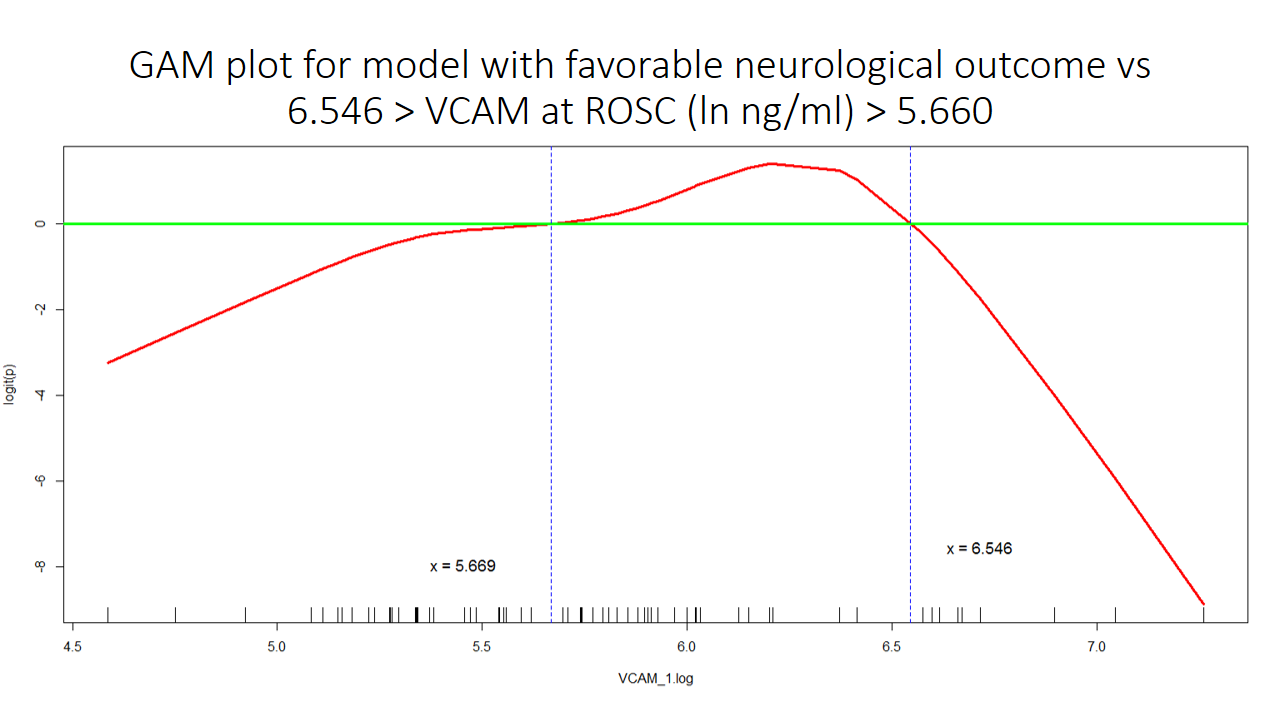


**Supplementary Figure S4:** Five-parametric fitting curves to convert median fluorescence intensities (MFIs) into concentration values for biomarkers.


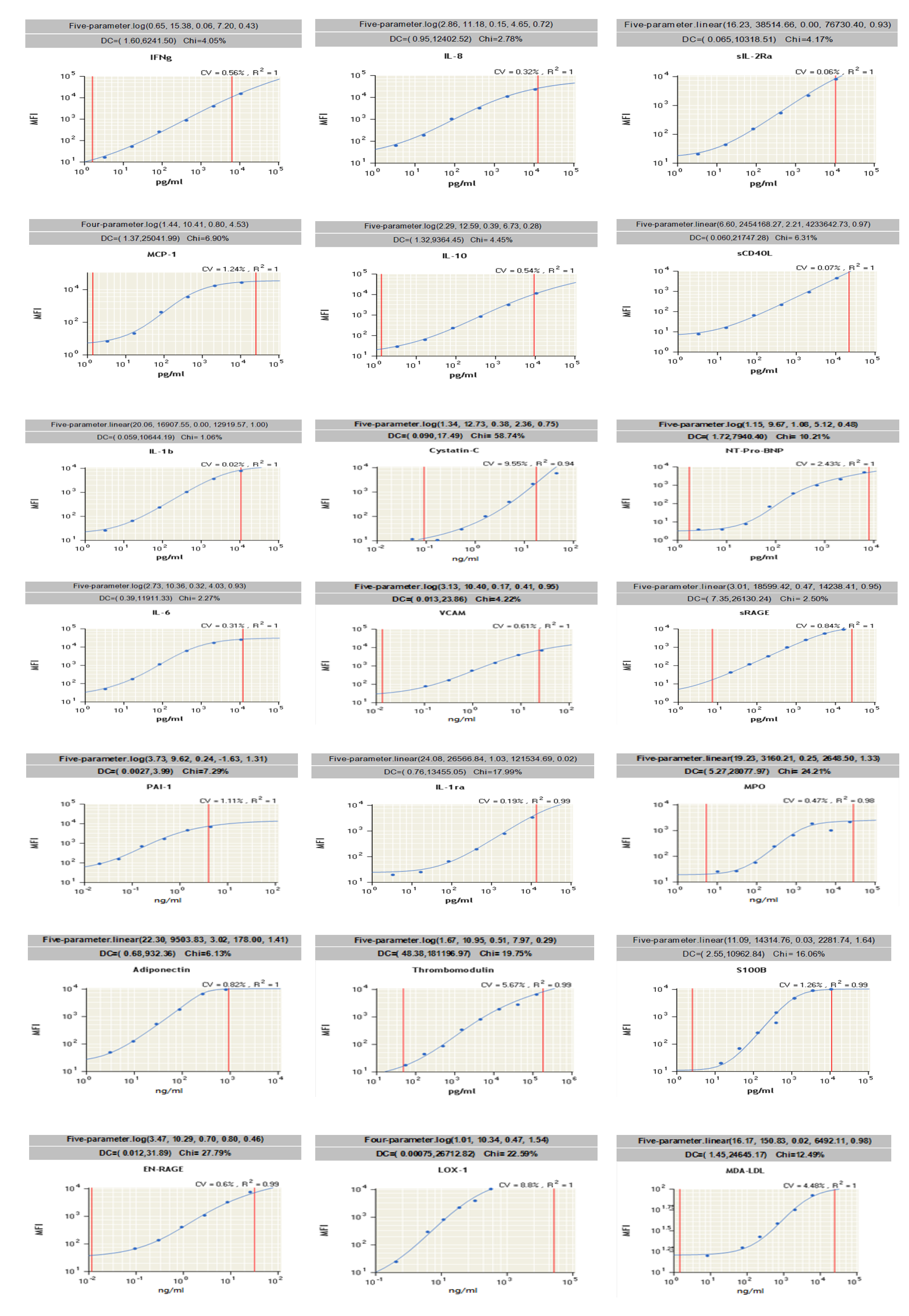

Supplement: Supplementary Information [file srep27187-s1.doc]
